# Predicting the Lay Preventive Strategies in Response to Avian Influenza from Perceptions of the Threat

**DOI:** 10.1371/journal.pone.0024943

**Published:** 2011-09-20

**Authors:** Jocelyn Raude, Michel Setbon

**Affiliations:** EHESP School of Public Health, Sorbonne Paris Cité, Rennes, France; Universidade Federal de Minas Gerais, Brazil

## Abstract

**Background:**

The identification of patterns of behaviors that lay people would engage in to protect themselves from the risk of infection in the case of avian influenza outbreak, as well as the lay perceptions of the threat that underlie these risk reduction strategies.

**Methodology/Principal Findings:**

A population-based survey (N = 1003) was conducted in 2008 to understand and describe how the French public might respond to a possible outbreak. Factor analyses highlighted three main categories of risk reduction strategies consisting of food quality assurance, food avoidance, and animal avoidance. In combination with the fear of contracting avian influenza, mental representations associated with the manifestation and/or transmission of the disease were found to significantly and systematically shape the behavioral responses to the perceived threat.

**Conclusions/Significance:**

This survey provides insight into the nature and predictors of the protective patterns that might be expected from the general public during a novel domestic outbreak of avian influenza.

## Introduction

During the last decade, the threat of avian influenza (AI) has received considerable attention from the scientific, political and lay communities around the world. Since the first diagnoses of highly pathogenic cases of H5N1 avian influenza in China, the virus has rapidly spread from Asia to Africa and Europe. To date, 564 human cases of avian influenza H5N1 have also been confirmed since 2003, mainly in Indonesia, Egypt and Vietnam, resulting in 330 deaths [1]. Despite vigorous disease control measures, influenza A viruses of subtypes H5 and H7 are still periodically discovered in wild and domestic birds. In France, several positive cases of H5N1 were found in wild duck on the national territory, and an outbreak eventually occurred in February of 2006 in a turkey farm in the Dombes Region. All birds from the farm were killed as a precautionary measure. These recurrent outbreaks have elicited extensive media coverage about the nature and causes of the threat and raised legitimate concerns about the risk to humans from multiple sources of infection in our country. Although there is scientific evidence that the majority of human cases of the disease were contracted following direct contact with infected animals, the possibility has not been excluded that the virus could be transmitted to humans through the consumption of improperly cooked poultry or poultry products [2]. Until the emergence of the pandemic A/H1N1 colloquially known as ‘swine’ flu in 2009, the increasing severity and magnitude of AI outbreaks had aroused the specter of a new and potentially devastating influenza pandemic – with comparable consequences to those of the Spanish flu (1918–19) [3]. Indeed, there was a major concern that the current highly pathogenic avian influenza viruses might mutate into more highly infectious forms for humans and acquire the ability of person to person transmission.

In recent years, the public's reaction to avian influenza has been investigated in many countries – including several Northern countries such as Italy, the United Kingdom, or the United States [4]–[6], and Southern countries such as Vietnam [7]. Indeed, it is increasingly recognized that the knowledge of how people will respond to the threat of avian influenza is critical to determine the potential epidemiologic and socioeconomic consequences of possible future outbreaks. Several studies have attempted to describe what proportion of individuals would take protective actions and which actions they would adopt. Empirical research on public response to the risk of contracting AI has shown that people undertook more or less adaptive behavioral responses to prevent the infection [8]. The most commonly reported measures for prevention of AI infection can be roughly divided into two categories. First, those minimizing the perceived exposure to secretions from potentially infected animals (for instance, avoiding contacts with surfaces or objects contaminated by feces from poultry or birds). Second, those reducing the perceived risks of infection from food-borne AI. Even if epidemiologic investigations showed that most cases of H5N1 virus transmission from birds to humans could be attributed to direct contact with live poultry [9], [10], the route of AI infection has long been remained controversial in the public arena, and the consumer response to the discovery of the H5N1 virus in poultry was immediate and massive, resulting in substantial declines in consumption in many countries [11]. However, it should be noted that avoidance of poultry does not represent the only possible measure of precaution. On the basis of the Roselius's pioneering work [12], Yeung and Morris [13] argued that individuals can adopt a range of coping strategies to decrease perceived risk in food consumption, mainly: (i) avoiding permanently or momentarily the consumption of potentially harmful products; (ii) reducing consumption of the potentially harmful product and, in so doing, reducing the perceived exposure to hazard; (iii) switching from certain types of product to others which are perceived safer, such as organic or origin labeled products; and (iv) continuing to consume the offending product and taking the risk of acquiring the disease.

To better understand the nature and the magnitude of the public's response to AI, risk perceptions have been extensively investigated [14]. Within the social and behavioral sciences, risk perception is generally conceptualized as the combination of two major components: the perceived likelihood of harm and the perceived severity of its consequences [15]. These components are assumed to motivate people to protect themselves from health risks. However, previous studies in the domain of emerging infectious respiratory diseases (SARS, avian influenza, pandemic influenza) have shown that the perceived risk was not strongly nor consistently associated with a range of behavioral changes. For example, in a large international survey conducted in 5 European and 3 Asiatic countries, Sadique et al. [16] found that risk perception was significantly associated with only one protective measure in response to the pandemic influenza threat and concluded that “neither the risk perception score nor its individual components seemed to affect preventative actions”. Similarly, Brug et al [17] found in Netherlands that the perceived risk had no statistical effect on a list of reported protective actions for SARS, while Lau et al. [18] showed in Hong-Kong that it only significantly predicted the avoidance of crowed places among a set of hygienic and social distancing measures, once the effects of sociodemographic and other confounding variables were controlled. These results are not very surprising since perceived risk is primarily defined as a motivating factor that may be moderated by a range of intermediate factors in most health behavior models [19]. Nevertheless, although there is still some disagreement about the strength (and relative importance) of the mechanism, it is generally assumed that perceptions of risk motivate individuals to adopt protective behaviors. In sum, the greater the perceived risk in terms of probability or severity, the greater is the intention to take action to reduce the risk.

From our viewpoint, two aspects of the public response to the AI threat deserve therefore more thorough analysis. First, it is not immediately apparent that adoption of particular protective measures to be a simple function of the perceived risk of contracting the disease even if it is clear that, where there is only one possible response to a health threat (e. g., smoking cessation for lung cancer prevention), studies of risk perception may be helpful to account for the public reaction to a health threat. However, in cases where there are more than one behavioral response, such as with AI, risk perception studies are undoubtedly insufficient to understand and predict the kind of protective actions preferred by individuals [20]. Overall, it seems fairly reasonable to assume that individuals make more complex cognitive works that lead them to adopt some kind of measures while rejecting others. Thus, it may be that some individuals are more likely than others to adopt particular risk reduction strategies once the risk of infection has been reported by the communication media.

Second, when faced to an emerging health threat people elaborate images and ideas about the nature and transmission of disease that are likely to be different across individuals. These cognitive elements – which have been more formally defined as “illness representation” or “illness perception” – have been argued to be the product of a series of psychological and sociological processes by which people select, filter, acquire, interpret, and alter information about the relative attributes of the disease [21]. Illness representations can be viewed as the mental base by which individuals develop strategies of health behavior. Evidence from many empirical studies provides strong support for a causal relation between the perception derived from these cognitive schemata and a range of more or less adaptive behavioral outcomes such as resistance to or compliance with public health recommendations [21].

Beyond the characterization of perceived risk, understanding factors that led populations to undertake one particular behavioral response rather than another could aid public health services to better deal with the repercussions and management of a possible novel outbreak of AI. Perhaps the most critical of these factors is the lay perception of the disease – its perceived transmission, manifestation and prevention, which are the focus of this paper. In recent decades, a vast array of empirical studies have shown that individuals and communities construct mental schemata or cognitive representations of health-threatening conditions that shape, to a large extent, the nature and performance of protective behaviors [21]. Among the most influential models of illness representations are those deriving from Moscovici's social representations theory [22] or Leventhal's common sense model [23]. Core concepts in almost all illness representation models incorporate *illness nature or identity* (i.e. what are the symptoms spontaneously attributed to the disease), *illness causes* (i.e., in the case of infectious diseases, the perceived route of transmission) and *illness prevention* (the perceived effectiveness of preventive/curative responses). These elements constitute the conceptual framework around which this study was constructed. If assumptions derived from illness perception theories are correct, the risk mitigation strategies undertaken by individuals may be at least partially the result of these mental representations. Having omitted these variables, previous research has not specifically tested for the effects of the factors on the adoption of a range of protective measures related to AI.

## Methods

To understand and predict how the French public might respond to a possible new outbreak of highly pathogenic avian influenza in either poultry or wild birds in Europe, we conducted a cross-sectional study of cognitive representations related to the threat of avian influenza [24]. The survey was conducting according to the principles expressed by the National Data Protection Authority (Commission Nationale Informatique et Libertés/CNIL) which is in charge of ethical issues and protection of individual data collection in France. However, the formal approval of this survey by the CNIL was not requested since the collection of anonymous and non-discriminatory public opinion data by professional survey companies is legally exempted from this procedure. Informed consent participant was orally obtained from the participants at the beginning of the interview after a thorough explanation of its purpose so that the data could be collected and analyzed anonymously.

### Participants

The primary data were collected in France by ED Institute by means of computer-assisted telephone interviews (CATI) of French adults aged 18 and over during June 2008. A proportional random digit dialing was used to select the survey participants across the country. To ensure the national representativeness of the sample, a stratified selection procedure based on the administrative area population (regions and communes/counties) was used. Furthermore, gender, age and occupational status of respondents were controlled by using quotas so that the sample approximated the last France Census data. As the sample did not differ from the whole population in education, size of household and socio-professional category by more than 2%, analyses were carried out using unweighted data. 37.3% of the households agreed to be interviewed, which can be regarded as a reasonable response rate when compared to previous studies performed on the same issue in western countries [8]. The mean time of questionnaire administration was 28 minutes. A total of 1003 participants completed the questionnaire.

### Measures

#### 1. Perception of risk

This concept has been demonstrated to present numerous dimensions that could potentially be investigated [25]. However, in line with leading theoretical frameworks in the field of health psychology – such as Rosenstock's Health Belief Model, Roger's Protection Motivation Theory, or Witte's Extended Parallel Processing Model – we propose to deal with only 3 of them: perceived severity (beliefs about the seriousness of the consequences of infection), perceived vulnerability (beliefs about the personal likelihood of becoming infected), and fear of the disease.

In our survey, the former construct (*perceived severity*) was assessed with a single question derived from the existing literature: “*How serious would it be for you to contract avian influenza*?” (scale of 0 to 10).

In the same vein, the *perceived vulnerability* was measured with a single question: “*How likely do you think it is that you contract avian influenza in the case of outbreak*” (scale of 0 to 100). To normalize the skewed distribution of the perceived vulnerability variable, a square-root transformation was made, which resulted in a measure on a scale from 0 to 10.

The latter construct (*fear of Avian Influenza*) was measured by using items from the Revised Illness Perception Questionnaire for Healthy people (IPQ-RH) developed by Figueras & Alves [26] (three items, Cronbach's alpha = 0.78, e.g., “*Thinking about avian influenza makes me feel afraid*”), with the wording adapted to fit with AI. In each case, participants were asked to select one of the five response options (“*strongly disagree*”, “*disagree*”, “*neither agree nor disagree*”, “*agree*”, “*strongly agree*”), which were scored on a scale of 1 to 5, with 1 indicating a strong disagreement and 5 a strong agreement.

#### 2. Perception of response efficacy

The perceived behavioral control and perceived efficacy of treatment were also examined in the survey. These variables are conceptually similar to those of efficacy and self-efficacy beliefs that have been extensively investigated in previous studies of perception of emerging infectious threat. They were measured by adapting again the IPQ-RH.

The *perceived behavioral control* variable examines whether people think that one can actually prevent the disease, and encompass three items (Cronbach's alpha = 0.64, e.g., “*The prevention of this disease depends on me*”).

The variable related to *perceived efficacy of treatment* examines whether people believe that the disease can be effectively cured or managed, and includes three items (Cronbach's alpha = 0.71, e.g., “*The negative effects of infection can be prevented by antiviral treatment*”).

All these questions were based on the same above-mentioned response format (scale 1–5). To make the various scores associated with the cognitive (fear of AI) and emotional variables (perceived behavioral control and efficacy of treatment) comparable, they were divided by the total number of items of the scale.

#### 3. Perception of illness

The items were developed specifically for the examination of the perception of avian influenza. Nevertheless, they were based on the phrasing used in the illness perception questionnaire [27], and adapted from focus group interviews conducted in the qualitative part of the study. These exploratory interviews were performed to investigate in-depth the schemes through which lay people interpret and understand the health risks associated with avian influenza. As the issues of transmission, manifestation and prevention of the disease were raised during the interviews, they could be discussed in more detail. The principal advantage of this qualitative method is that people can express their opinions and views in terms of ideas or wordings that are not necessarily those of the researchers. The portion of the survey devoted to mental representations of AI was divided into 2 main sections.

In the first section, a disease identity scale related to *clinical manifestation*: was presented with 9 symptoms (e.g., *sudden fever*, *diarrhea*, *vomiting*, *cough*, *respiratory distress*, etc.) that are erroneously or properly attributed to the disease. Participants were asked whether or not they believed the symptom to be related to AI infection (yes/no).

The second section devoted to *modes of transmission* addressed the perceived routes of transmission of the AI infection. Respondents were given a list of bird-related materials (6 items, e.g., *feces from infected birds*, *respiratory secretions from infected birds*, *cooked meat from infected poultry*, *raw eggs from infected poultry*, etc.) and asked whether they thought that these materials constituted a possible route of transmission of AI virus (yes/no).

#### 4. Protective behaviors

In the last section, participants were given a list of 11 protective actions recommended or observed by the public health authorities in France during the 2006 outbreak (e.g., *avoiding direct contact with objects spoiled by birds feces*, *avoiding contact with live or dead wild birds*, *eating only properly cooked meat from chicken*, *avoiding consumption of raw eggs from chicken*, etc.) and asked whether or not they would take any of them to reduce the risk of infection (yes/no) in case of AI outbreak.

The questionnaire also included a large range of items which aimed to collect socioeconomic and demographic information on the participants (age, gender, education, family income, marital status, work status, occupational status and size of household).

### Analytic strategy

To explore the structure of the cognitive representations of AI infection and determine which of the items may be grouped into categories of perceived symptoms, routes of infection, and means of precaution, separate multiple correspondence analyses (MCA) were conducted on the data collected from the 1003 individuals. MCA is a factor analysis method designed to examine relationships among nominal or categorical variables by summarizing them into a smaller number of orthogonal variables called principal components [28]–[29]. MCA is an extension of simple correspondence analysis that allows for the graphical representation of statistical association between the responses to a set of categorical variables in a lower Euclidian dimensional space, in order to uncover the underlying dimensions best able to describe the main oppositions or associations in the data. From the indicator matrix, MCA isolates a certain number of axes, each of which scatters the binary responses along one dimension. The eigenvalues calculated for each different axis permit us to assess the amount of variance explained by each axis and therefore the quality and accuracy of the graphical representation. MCA can be considered as a generalization of principal component analysis when the measurements to be analyzed are categorical instead quantitative. The interpretation in MCA is generally based upon proximities between points associated with modalities in the multi-dimensional map. The more the responses to the different binary questions tend to be observed together, the more these responses are located close to others in the low-dimension space [30]. Thus, if everyone who reported avoidance of cooked poultry also reported avoidance of cooked eggs, these modalities would be located in the same position. The objective of this factor analysis was to highlight the cognitive schemata that transcend particular beliefs and behaviors in response to the AI threat.

Participants' responses were then summed across items to generate subscales related to the manifestation, transmission or prevention of the disease, so that a higher score represents a stronger endorsement of the constructs. The scores obtained for the prevention-related constructs were then recoded as dummy variables: less than the mid-scale value (coded as 0), equal or higher than the midscale value (coded as 1). In the same vein, data from the 5-point Likert items used to evaluate the perceptions of risk and perceptions of response efficacy were reduced to the nominal level by combining the positive options (“*strongly agree*”, “*agree*”) on the one hand, and the negative options on the other hand (“*strongly disagree*”, “*disagree*”, “*neither agree nor disagree*”) into two categories of “disagree” (coded as 0) and “agree” (coded as 1). They were then summed to generate a score for each group of items.

Finally, a series of logistic regression model was used to assess the influence of the cognitive representations of the threat on the various risk reduction strategies. The dependent variables were to what extent the participants were likely to undertake one particular type of risk reduction strategy revealed by the MCA. For each strategy, the explanatory variables were the different subscales related to the perceived clinical manifestations and routes of transmission of the disease, as well as the range of common predictors related to individuals' perceptions of risk and response efficacy. Participants' age, gender, education, and occupational status were controlled for the regression analyses. The statistical analyses were performed with either STATA (version 10) or SPSS (version 13).

## Results

### Perceived risk of avian influenza

The avian influenza fear scale was normally distributed with an average score of 3.08 (SD = 1.38; IQR = 2–4). More than half of the participants (59.7%) had a score below or equal to 3 (the midpoint of the scale) indicating that they didn't fear the disease. Severity scores were positively skewed, with an average score above the midpoint of the scale (mean = 6.72; SD = 2.25; IQR = 5–8), whereas vulnerability was negatively skewed with an average score of 4.65 (SD = 2.66; IQR = 2.2–7.1) on a scale of 0 to 10.

### Perceived efficacy of response

The perceived behavioral control scale was positively skewed, with an average score of 4.18 (SD = 0.95; IQR = 4–5). About three-quarters of the participants (76%) had a score above 3, indicating that the majority of the population thought that avian influenza could be effectively prevented by certain protective measures. By contrast, the perceived effectiveness of pharmaceutical treatments was normally distributed (mean = 3.27; SD = 0.68).

### Perceived clinical manifestation

**Figure 1:**
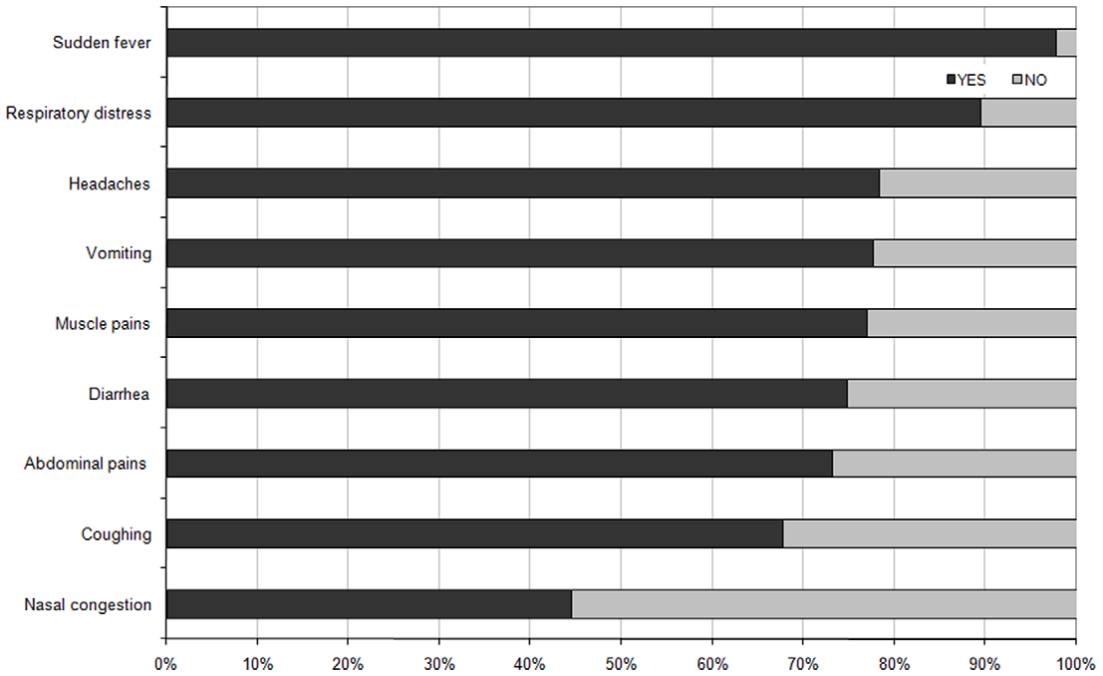
Percentage of respondents who believed that the symptom might result from an AI infection.

**Figure 2:**
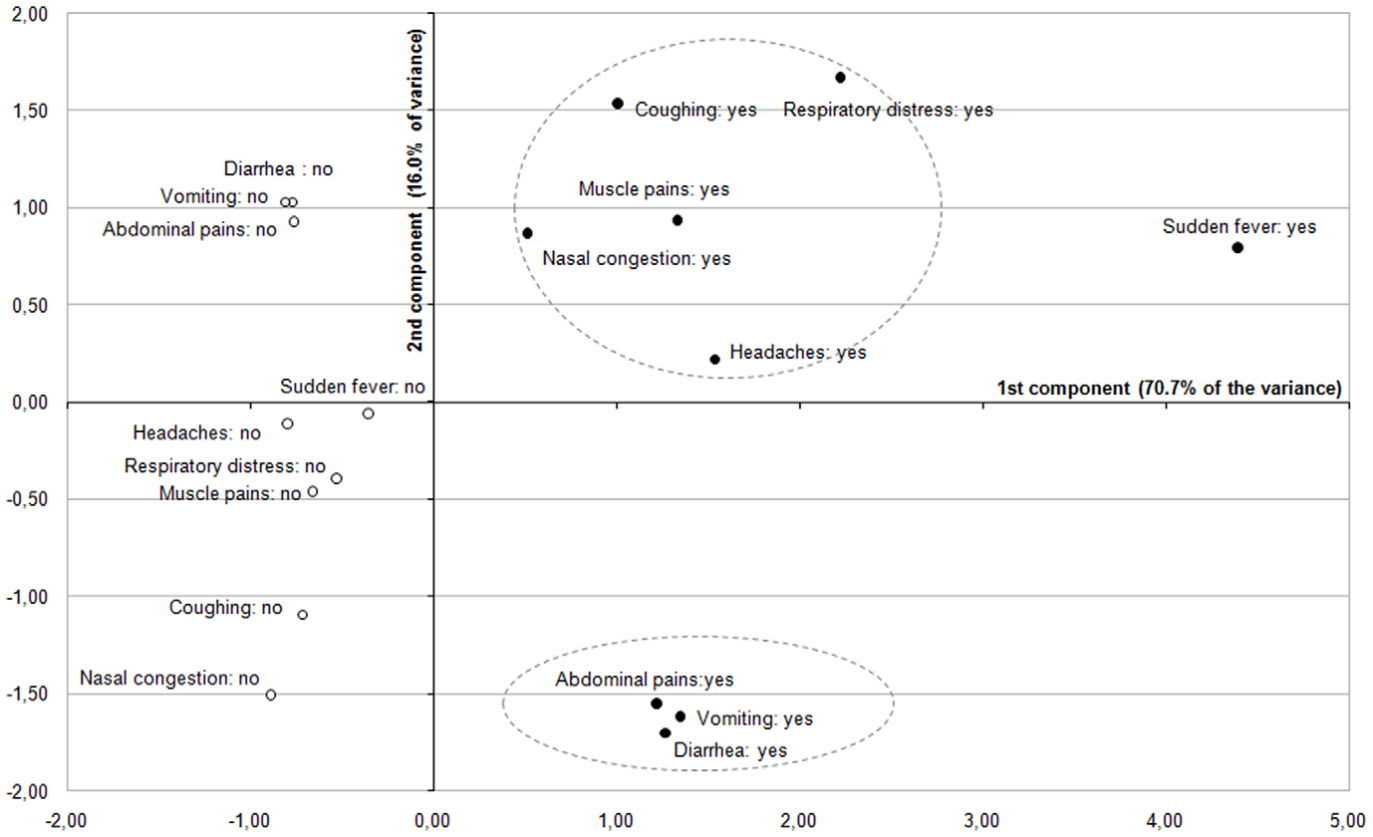
Multiple correspondence analysis of the symptom-related items (first and second principal components). Each item is visualized with a point: a black circle for ‘positive’ response categories (•), and an empty circle for the ‘negative’ response categories (○).

We first investigated the frequencies with which the different AI symptoms were identified by the participants (Figure 1). With the notable exception of *nasal congestion*, a majority of participants reported that all these symptoms could be associated with an AI infection, indicating a certain degree of confusion in the perception of the nature of the disease. Nevertheless, *sudden fever* and *respiratory distress* – which have long been recognized as typical AI infection signs in the biomedical sciences – were the most frequently identified symptoms (>80%). The remaining symptoms were identified as relevant AI clinical manifestations by more than half of the respondents. Then, multiple correspondence analysis was conducted to construct summed rating subscales. This analysis produced a 2-factor solution which together account for about 87% of the variance (Figure 2). The first component displays the yes-saying responses associated with *coughing*, *headaches*, *nasal congestion*, *muscle pains*, *respiratory distress*, *diarrhea*, *abdominal pains* and *vomiting* at the same level in the positive values (on the right-hand side of the space). The second component opposed *headaches*, *nasal congestion*, *muscle pains coughing*, *sudden fever* and *respiratory distress* in the positive values, and *diarrhea*, *abdominal pains* and *vomiting* in the negative values. Overall, MCA allows distinguishing between 2 clusters of clinical manifestations associated with an AI infection that can be easily interpreted as food poisoning-like symptoms (on the bottom right quadrant of the graph), and flu/pneumonia-like symptoms (on the top right quadrant of the graph). Both subscales showed an acceptable internal consistency (with alpha coefficients of 0.82 and 0.65, respectively). The former was positively skewed, with an average score of 2.39 (SD = 0.95; IQR = 2–3), whereas the latter was rather normally distributed, with an average score of 3.08 (SD = 1.45; IQR = 2–4).

### Perceived routes of transmission

**Figure 3:**
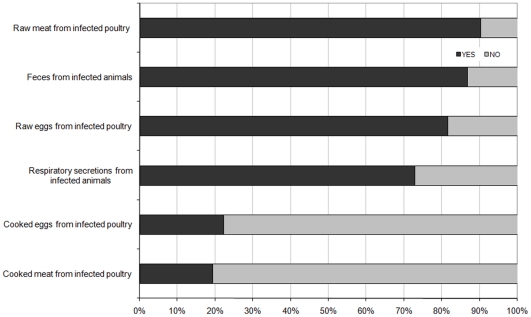
Percentage of respondents who believed that the object represents a possible route of AI transmission.

**Figure 4:**
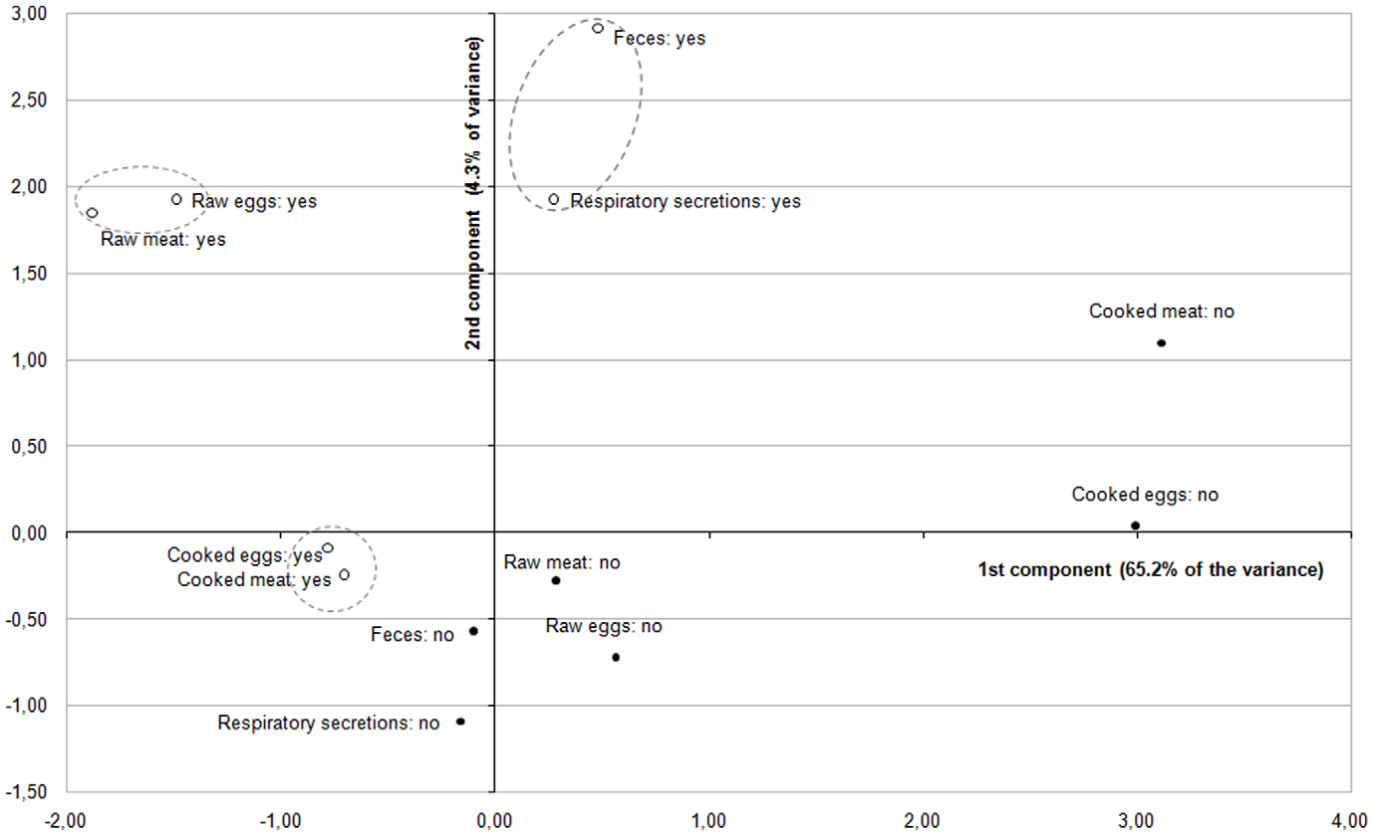
Multiple correspondence analysis of the transmission-related items (first and second principal components). Each item is visualized with a point: a black circle for ‘positive’ response categories (•), and an empty circle for the ‘negative’ response categories (○).

Although there is currently little epidemiological evidence suggesting that people have been contaminated through the consumption of products from infected poultry, *raw meat* was the most widely reported route of AI infection by the respondents (>86%). Objects or surfaces contaminated by feces, respiratory secretion, and raw eggs from infected animals were also considered to be possible infection routes by a majority of participants (Figure 3). In contrast, consistent with the biomedical literature, cooked poultry and egg products were rarely identified as materials with potential risk for acquiring AI infection (<21%). MCA was again used to analyze the data Factor analysis leads to reveal a two principal components structure explaining 69% of the variance (Figure 4). The first identified component opposed along the axis 1 items associated with cooked and raw food products (*eggs* and *poultry*) on the left-hand side to those associated with animal excretions (*respiratory secretions* and *feces*) in the right-hand side. The second component mostly opposed along the axis 2 the items associated with the animal excretions (*respiratory secretions* and *feces*) and those associated with raw food products (raw eggs and poultry) on the top quadrants to the items related to the cooked food products (cooked *eggs* and *poultry*) on the bottom left quadrant. Thus, the 3 clusters that emerged from the MCA were relatively easy to interpret as discriminating elements associated with raw products, cooked products, and animal excretions. However, Cronbach's alphas were acceptable for both subscales related to food items (cooked products *versus* raw products: α = 0.82 and 0.62, respectively) but not for the subscale related to animal excretions. Since the association between these two items demonstrated an insufficient internal consistency (α = 0.36), *respiratory secretions* and *feces* were not grouped but introduced separately in the regression models. The subscale related to raw products was positively skewed, with an average score of 1.59 (SD = 0.25; IQR = 1–2), while that related to cooked products was negatively skewed, with an average score of 0.25 (SD = 0.43; IQR = 0–1).

### Measures of protection

**Figure 5:**
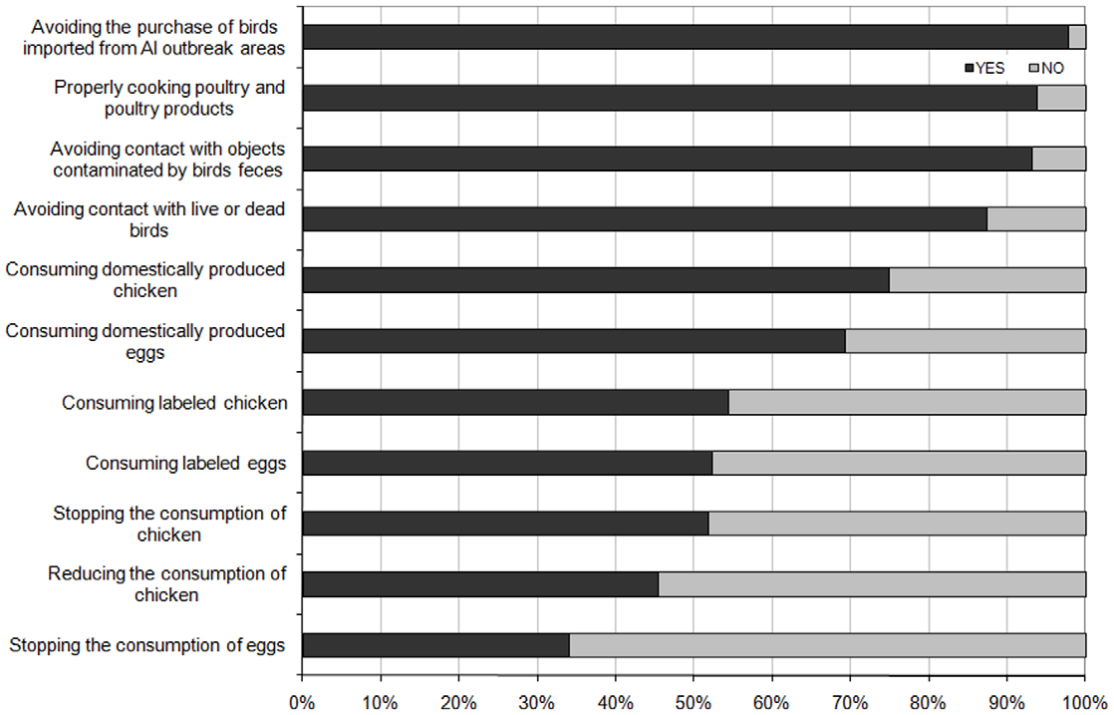
Percentage of respondents who reported that they would take the protective action in case of domestic outbreak.

The protection scale included a large range of risk reduction measures – from avoidance of dead or live birds to the consumption of labeled poultry products. Overall, the protective measures that require no or small behavioral change were found to be more relevant to people than those that require more significant behavioral change, notably in terms of food consumption (Figure 5). For instance, more than 92% of participants reported that they would avoid direct contacts with surfaces and objects contaminated by bird feces, while only about 45% declared that they would be likely to reduce their chicken consumption. On the basis of McNemar's test of all pairs of these two categories using a *p*<0.05 criterion, all pairings were found significantly different with the notable exception of *avoiding contacts with contaminated objects* versus *properly cooking poultry*.

**Figure 6:**
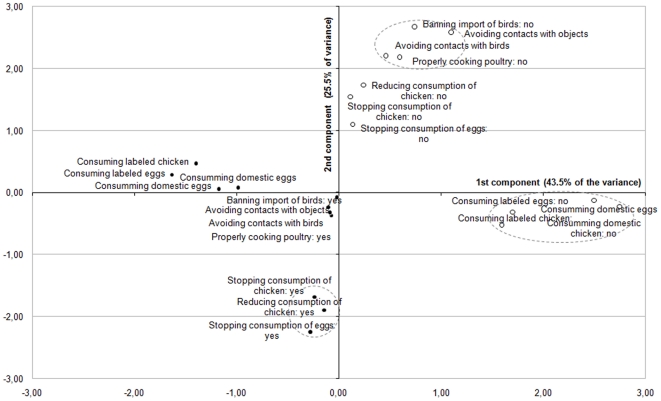
Multiple correspondence analysis of the prevention-related items (first and second principal components). Each item is visualized with a point: a black circle for ‘positive’ response categories (•), and an empty circle for the ‘negative’ response categories (○).

**Figure 7:**
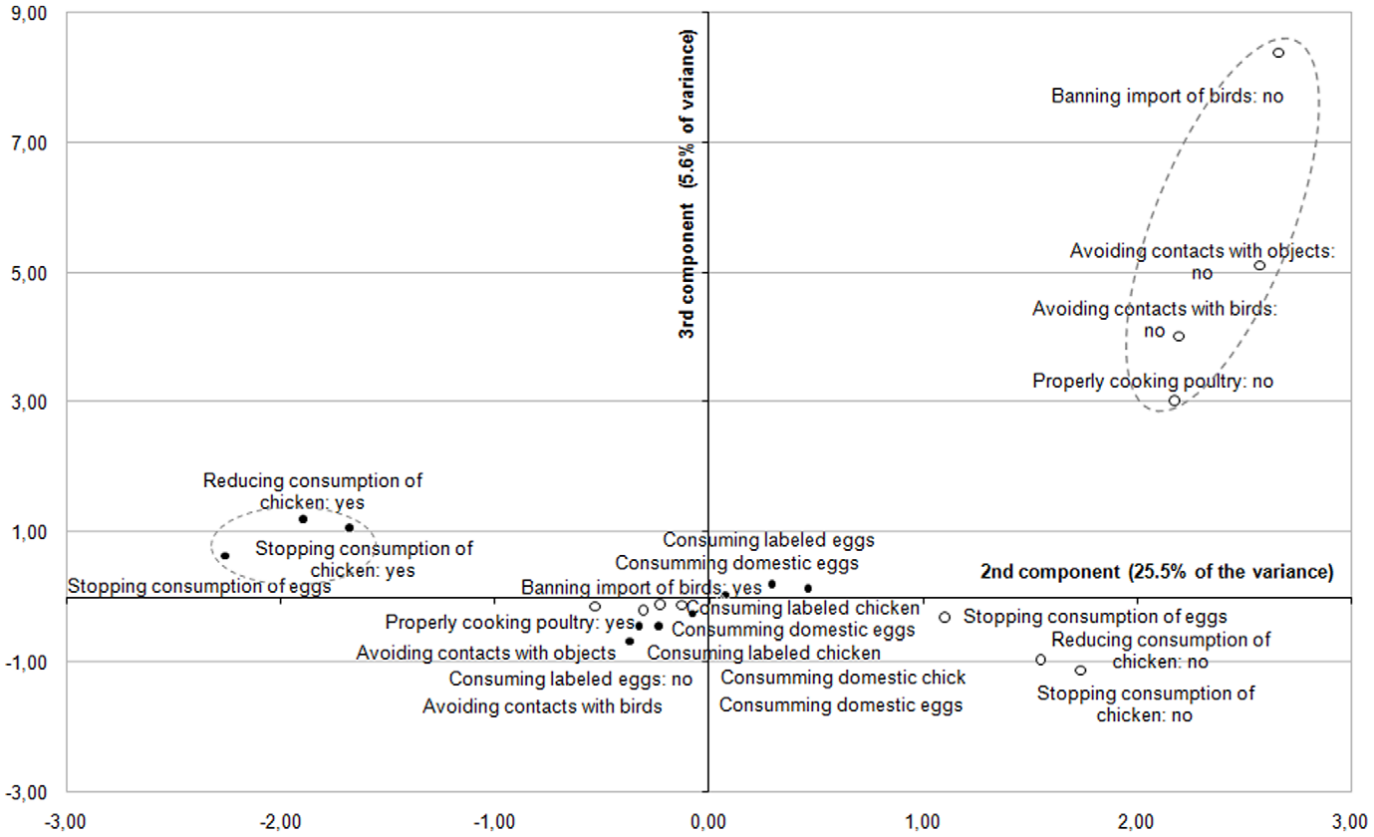
Multiple correspondence analysis of the prevention-related items (second and third principal components). Each item is visualized with a point: a black circle for ‘positive’ response categories (•), and an empty circle for the ‘negative’ response categories (○).

A MCA was finally performed to investigate the structure of the protective behavior in response to AI and identify the main strategies adopted by respondents. This factor analysis produced a 3-principal components solution accounting for approximately 75% of the variance. A look at the Figure 6 reveals that, on axis 1, most of the positive modalities are located on the left-hand side, and the negative modalities are located on the middle or the right-hand side. The second component mostly opposed the positive modalities associated with qualitative change in the food consumption (consuming *labeled* or *domestic* food products) in the bottom right quadrant, to the other positive modalities in the top right quadrant. By contrast, if one look at the Figure 7, one can find a cluster of four negative modalities mostly associated with avoidance measures (e.g. *avoiding direct contact with objects spoiled by birds' feces*) in the top right quadrant, while the others modalities are all located near to the middle on axis 3. Although the Figures 6 and 7 drawn from the MCA are a bit more complicated to interpret because of the number of modalities examined, their results tend to reveal three distinctive risk reduction strategies among lay people that could be labeled as food quality assurance (I), food avoidance (II), and animal avoidance (III). All the subscales demonstrated an acceptable reliability, with Cronbach's alpha coefficients ranging from 0.79 for the food quality assurance to 0.56 for the animal avoidance measures. These results tend to support to a large extent the Roselius's conceptual framework [12], [13], although the total avoidance measures were not found to be separated by the respondents from the partial avoidance measures in the case of avian influenza.

### Prediction of preventive strategies from perceptions

Multivariate logistic regression analyses were then performed to model the association between the different types of protective actions that were reported in response to AI and the mental representations related to the manifestation and transmission of the disease, as well as the variables associated with perceived risk of AI infection and efficacy of responses. The coefficients in Table 1 to [Table pone-0024943-t002] 3 represent the beta-values in the logistic regression equations. Consistent with previous studies, the emotional arousal caused by the threat, i.e. *fear of contracting the disease*, was found to be a strong predictor of health protective behaviors across a range of risk reduction strategies. The other variables introduced in the regression models – perceived risk (severity and vulnerability), perceived efficacy (behavioral control and effectiveness of treatment), and socio-demographic factors – showed no or inconsistent effects on the adoption of protective actions. For instance, the perceived severity, behavioral control and effectiveness of treatment impacted significantly no more than one risk reduction strategy in multivariate analysis. Thus, only the emotional component was found to systematically motivate protective action.

**Table 1:** Logistic regression analyses for prediction of the food quality strategy: unadjusted and adjusted odds-ratio (95% CI).

	Unadjusted odds-ratio	p-value	Adjusted odds-ratio*	p-value
***Perceived risk***				
Fear	**1.204 (1.067–1.358)**	**.003**	**1.188 (1.050–1.345)**	**.006**
Severity	**.765 (.626–.935)**	**.009**	**.758 (.617–.932)**	**.009**
Vulnerability	1.269 (.982–1.639)	.069	1.257 (.968–1.633)	.086
***Perceived efficacy***				
Control	.932 (.813–1.067)	.307	.982 (.853–1.130)	.796
Treatment	**1.564 (1.198–2.042)**	**.001**	**1.472 (1.120–1.934)**	**.006**
***Perceived clinical manifestations***				
Poisoning-like symptoms	**1.438 (1.096–1.886)**	**.009**	**1.335 (1.009–1.765)**	**.043**
Pneumonia-like symptoms	1.066 (.973–1.167)	.171	1.055 (.961–1.159)	.260
***Perceived routes of transmission***				
Feces	.894 (.604–1.324)	.577	.857 (.574–1.281)	.452
Respiratory secretions	1.275 (.935–1.738)	.125	1.251 (.911–1.718)	.166
Raw products	1.067 (.867–1.314)	.540	1.080 (.862–1.337)	.481
Cooked products	1.289 (.951–1.747)	.102	1.233 (.900–1.688)	.192
**Nagelkerke's pseudo-R^2^**	**6.0%**		**10.3%**	

*Adjusted for age, sex, occupation, and level of education.

**Table 2 pone-0024943-t002:** Logistic regression analyses for prediction of the food avoidance strategy: unadjusted and adjusted odds-ratio (95% CI).

	Unadjusted odds-ratio	p-value	Adjusted odds-ratio*	p-value
***Perceived risk***				
Fear	**1.378 (1.242–1.529)**	**.000**	**1.405 1.262**–**1.563)**	**.000**
Severity	.955 (.775–1.175)	.661	.925 (.748–1.143)	.469
Vulnerability	1.131 (.936–1.368)	.202	1.136 (.937–1.377)	.193
***Perceived efficacy***				
Control	.952 (.826–1.098)	.502	.965 (.937–1.377)	.629
Treatment	**1.269 (1.037–1.552)**	**.021**	**1.273 (1.038–1.561)**	**.021**
***Perceived clinical manifestations***				
Poisoning-like symptoms	1.143 (.856–1.526)	.364	1.089 (.810–1.465)	.573
Pneumonia-like symptoms	1.092 (.950–1.256)	.217	1.085 (.833–1.116)	.267
***Perceived routes of transmission***				
Feces	1.204 (.803–1.806)	.368	1.135 (.940–1.252)	.546
Respiratory secretions	.916 (.667–1.258)	.588	.876 (.634–1.211)	.424
Raw products	1.234 (.990–1.538)	.061	1.239 (.990–1.549)	.061
Cooked products	**1.383 (1.145–1.669)**	**.001**	**1.388 (1.145–1.684)**	**.001**
**Nagelkerke's pseudo-R^2^**	**10.7%**		**12.8%**	

*Adjusted for age, sex, occupation, and level of education.

**Table 3:** Logistic regression analyses for prediction of the animal avoidance strategy: unadjusted and adjusted odds-ratio (95% CI in parentheses).

	Unadjusted odds-ratio	p-value	Adjusted odds-ratio*	p-value
***Perceived risk***				
Fear	**1.260 (1.056–1.505)**	**.010**	**1.230 (1.027–1.473)**	**.024**
Severity	.872 (.702–1.084)	.217	.879 (.706–1.095)	.251
Vulnerability	.843 (.681–1.044)	.118	.840 (.676–1.045)	.118
***Perceived efficacy***				
Control	**.812 (.670–.983)**	**.033**	**.803 (.659–.977)**	**.028**
Treatment	.791 (.545–1.147)	.216	.759 (.520–1.108)	.153
***Perceived clinical manifestations***				
Poisoning-like symptoms	1.370 (.941–1.995)	.100	1.334 (.910–1.956)	.140
Pneumonia-like symptoms	**1.833 (1.254–2.680)**	**.002**	**1.815 (1.228–2.682)**	**.003**
***Perceived routes of transmission***				
Feces	**2.112 (1.326–3.365)**	**.002**	**1.968 (1.226–3.160)**	**.005**
Respiratory secretions	**1.870 (1.261–2.773)**	**.002**	**1.887 (1.261–2.823)**	**.002**
Raw products	1.124 (.839–1.505)	.434	1.129 (.839–1.519)	.424
Cooked products	.888 (.585–1.350)	.579	.840 (.548–1.285)	.421
**Nagelkerke's pseudo-R^2^**	**11.8%**		**13.2%**	

*Adjusted for age, sex, occupation, and level of education.

However, the perceived clinical manifestations and transmission routes related to AI were found to selectively influence the nature of the precautions that participants would undertake in case of a domestic outbreak (although the representations of routes of transmission did not significantly impact all the risk reduction strategies in multivariate analyses). Overall, respondents who thought that AI symptoms are similar to those of food poisoning were more likely to adopt protective measures which have been interpreted as food quality assurance. Symmetrically, people who believed that AI and seasonal influenza infection produced analogous clinical manifestations were more likely to avoid direct contact with wild or domestic animals. In the same vein, respondents who thought that the disease could be transmitted through the consumption of raw or cooked products were more likely to adopt protective behavior related to food, while those who believed that AI spread by direct contact with infected poultry (or objects/surfaces contaminated by their feces) had a higher inclination to take protective actions leading to avoidance of potentially infected animals and their excretions.

## Discussion

The objective of this paper was to better understand and predict the response of the lay public to a novel outbreak. Identifying the behavioral changes that might be expected in the face of an epizootic, as well as the cognitive factors that lead to specific preventive strategies, might significantly help the public health authorities to improve their risk communication and management strategies. To describe the nature of the public response to the perceived threat, we first investigated the health protective behavior that participants would take in case of a novel outbreak. Our results indicate that a majority of persons would be likely to undertake behaviors to reduce the risk of contracting the disease, although considerable differences were observed among the types of reported actions. Overall, the measures requiring small behavioral change, such as avoidance of contacts with potentially infected materials, appeared more relevant to people than those requiring larger behavioral change to reduce the perceived exposure to virus, through the consumption of potentially infected poultry products. Participants were likely to report their intention to practice a range of behaviors that are already performed, to a large extent, for other reasons. This lead us to conclude that the adoption of health protective measures tend to be facilitated if the interventions promoted by the public authorities only consist in activating, maintaining or reinforcing pre-existent practices in the case of an outbreak. It should also be noted that this pattern of protective actions was rather congruent with those found in the recent empirical studies conducted in various countries – including the United Kingdom [5], the United-States [6], and Vietnam [7]. After having described what proportion of persons would take what protective measures, we also attempted, by using factor analysis, to identify the main risk reduction strategies that would be employed by the participants in the face of a potential outbreak of AI. In the matter of food-borne risk, previous work suggested that laypeople might selectively cope with the risk by stopping, reducing or modifying their consumption of the risky products. Our results showed that people tend principally to distinguish between two risk reduction strategies: assurance of product quality and avoidance of poultry products. Although the total and partial avoidance measures were not found to be differentiated by respondents, the distinction between the quality-oriented and quantity-oriented precautions in response to the food-borne risk proposed for the first time by Roselius in 1971 was largely confirmed. However, it appeared that people were more likely to adopt the first risk reduction strategy than the second one; even if there was no scientific evidence demonstrating that labeled poultry products are safer than other poultry products in the case of AI outbreak.

To account for the manner in which people reduce their exposure to the perceived risk of AI infection, we tested a behavioral model based on the mental representations of the threat. Since risk perception models were found to be of limited interest in these circumstances (i.e., *necessary but not sufficient*), we also attempted to identify the mental schemata that underlie the risk reduction strategies adopted by laypeople. During recent decades, a large number of empirical studies have shown that cognitive representations directly guide the selection and performance of procedures for preventing or controlling infectious diseases such as AIDS or tuberculosis [21]. In the case of AI, the statistical analyses provided insightful and promising results that lead us to represent the precaution adoption process as the selection of alternative strategies of protective behaviors, while numerous prominent health behavior models assume shifts from inaction to action explained by difference in the value of continuous variables. Noticeably, the emotional covariable (*fear of avian influenza*) was found to motivate people to reduce the risk of infection regardless the nature of health protective behaviors, while perceptions of manifestation and transmission of the disease were found to orientate the choice of the risk reduction strategy they would favor in the case of a novel outbreak. Nevertheless, multivariate analyses showed that statistical influence of these representations on the risk reduction strategies potentially undertaken by individuals was significant but rather moderate. As indicated in tables 1 to [Table pone-0024943-t002] 3, Nagelkerke's Pseudo-R^2^ coefficients only ranged from 10.3% for the food quality assurance measures to 13.2% for the animal avoidance measures. Thus, it should be noted that the largest part of the intention to adopt one particular preventive strategy in the case of an AI outbreak was not explained by the independent variables explored in our survey, regardless their combination. As we mainly focused on intra-personal variables in this study, it is possible that other factors which were not considered in the study (e.g. social influence and norms, through the encouragement of relatives or health professionals) might play a considerable role in the perceived relevance of these various health protective behaviors.

Beyond the limited power of explanation of the regression equation, the key finding was from our viewpoint that each cognitive representation was significantly associated with distinct types of measures that the participants would take to reduce the risk of contracting the disease (with the only exception being that of the perception of animal excretions as route of transmission that lack any variance) and that the pattern of behavioral response was compatible with that predicted by the model. For example, the data showed that the more the participants believed that cooked products might be a possible route of infection, the more likely they were to report they would not eat chicken and eggs in the case of a domestic AI outbreak. Finally, these results tend to be congruent with the heuristic of symmetry that has been presented in previous research on the self-regulation of health threat [31]. Indeed, the perceptions of the threat were found to significantly trigger risk reduction strategies that “fit” with these representations, even though the beta-coefficients in the regression equation appeared somewhat moderate. Nevertheless, it should be noted that predictive studies in health behavior research have typically been found to explain small amounts of variance of reported health protective behavior, even when they address a much larger range of psychosocial factors [19].

To conclude, it is important to note that outcomes of this research are subject to a degree of uncertainty, due to the hypothetical nature of our questions. Numerous works have demonstrated that protective behaviors significantly diverge from what people think they will be in an effective situation. Nevertheless, the immediate and substantial change in the patterns of consumption observed in France in February 2006 after the discovery of the first H5N1 case in domestic birds provides empirical support to our findings. What consequence might be expected from the behavioral response to a massive outbreak of AI in France today? At this time, it seems to us that two hypotheses could be reasonably advanced. The first hypothesis is that the social experience of the A/H1N1 pandemic influenza has dramatically and durably changed the attitudes of European populations toward emerging respiratory infectious diseases. This could mean that a certain degree of saturation may have already been achieved regarding the media and public's potential attention for new health threats. Therefore, the detection of new cases of AI by the veterinary surveillance networks might not trigger any substantial behavioral change in France. The second hypothesis is that Europeans now clearly distinguish between the A/H1N1 pandemic influenza – which was largely perceived as a mild disease during the peak of the epidemic – and avian influenza, which would continue to be primarily viewed as a highly pathogenic disease that has preserved its frightening power.

In this hypothesis, the results of this survey suggest that a large majority of people would possibly take appropriate actions to reduce the risk of infection either through minimizing direct contact with infected birds and their feces, or by avoiding the consumption of improperly cooked meat of infected poultry. However, the data also show that about half would be likely to reduce the threat by rejecting most poultry products and/or by modifying their pattern of poultry consumption. Clearly, these strategies constitute maladaptive responses since the probability of infected poultry or eggs entering the food chain – whatever their nature (conventional, organic or certified food products) – would be extremely low in European Union countries. Moreover, the social and economic cost of a massive avoidance of poultry consumption, as key public response to the perceived risk of contracting the disease, could be potentially catastrophic since chicken represents a traditional food which is appreciated in most French households.
